# Does social support always help? Emotion regulation pathways through self-compassion in maltreated adolescents’ PTSD and growth

**DOI:** 10.3389/fpsyt.2026.1889693

**Published:** 2026-08-27

**Authors:** Qingyun Yu, Peizhong Wang, Tianchan Shi, Li Wang, Juncheng Guo, Wenchao Wang

**Affiliations:** 1 Normal College, Jingchu University of Technology, Jingmen, China; 2 Research Team of Integrated Mental Health Education for Primary, Secondary, and Higher Education, Jingchu University of Technology, Jingmen, China; 3 Beijing Key Laboratory of Applied Experimental Psychology, Faculty of Psychology, Beijing Normal University, Beijing, China

**Keywords:** adolescents, childhood maltreatment, emotion regulation, posttraumatic growth, posttraumatic stress disorder, self-compassion, social support

## Abstract

**Background:**

Adolescents exposed to childhood maltreatment often experience co-occurring posttraumatic stress disorder (PTSD) and posttraumatic growth (PTG). Perceived social support may influence these outcomes through emotion regulation strategies, yet its effects can be both protective and paradoxical. Self-compassion, comprising positive and negative dimensions, has been conceptualized as an emotion regulation strategy that may mediate these relationships.

**Methods:**

A two-wave longitudinal design with a six-month interval was employed. Participants were 1,773 Chinese adolescents (52.3% female) who reported childhood maltreatment. At Time 1, perceived social support, self-compassion, PTSD, and PTG were assessed; at Time 2, self-compassion, PTSD, and PTG were re-assessed. Structural equation modeling was used to test the hypothesized dual-pathway mediation mode.

**Results:**

Perceived social support positively predicted both positive self-compassion and negative self-compassion. Positive self-compassion was associated with lower PTSD and higher PTG, whereas negative self-compassion predicted only higher PTSD. Significant indirect effects indicated that social support was associated with reduced PTSD and increased PTG through positive self-compassion. Moreover, social support may be marginally associated with elevated PTSD through negative self-compassion in a potential maladaptive pathway.

**Conclusion:**

Findings reveal a dual-edged role of perceived social support among maltreated adolescents, operating through distinct self-compassion pathways. The adaptive pathway via positive self-compassion is associated with recovery and growth, while the potential maladaptive pathway via negative self-compassion may be associated with elevated distress. These results highlight self-compassion as a critical emotion regulation mechanism linking social resources to posttraumatic outcomes and suggest that interventions fostering positive self-compassion while addressing self-criticism could benefit trauma-exposed youth.

## 1 Introduction

### 1.1 Adolescence as a critical period for trauma exposure and posttraumatic reactions

Adolescence is a developmental stage marked by profound neurobiological, psychological, and social reorganization (1). During this period, individuals face heightened sensitivity to environmental stressors and an elevated risk of exposure to traumatic events (2). The family, as the primary context for socialization and emotional development, plays a foundational role in shaping adolescents’ self-concept and psychological adjustment (3). Epidemiological research has consistently documented a high prevalence of trauma exposure among adolescents worldwide. A recent systematic review reported that approximately 25% of trauma-exposed children develop posttraumatic stress disorder (PTSD) before reaching 18 years of age (4). Among Chinese adolescents, the picture is similarly concerning: one study among sixth-grade students found a PTSD positive screening rate of 7.48%, with childhood maltreatment—particularly emotional abuse and physical neglect—emerging as significant risk factors (5). The vulnerability of adolescents to trauma is compounded by the fact that emotion regulation capacities, which are critical for adaptive coping, continue to develop throughout this period and are not yet fully mature (6, 7). The developmental reorganization of emotion regulation during adolescence is further underscored by recent theoretical work proposing a ‘social reorientation’ of emotion regulation, wherein the primary regulatory functions shift from family to peer relationships across this developmental period (8). For adolescents whose primary maltreatment occurred within the family system, this reorientation may be particularly consequential, as the very individuals who constitute their traditional support network may also be the source of trauma. As such, adolescence represents both a window of heightened risk for trauma-related psychopathology and a window of opportunity for the development of adaptive emotion regulation skills that can shape long-term mental health trajectories.

Among the various forms of trauma to which adolescents are exposed, childhood maltreatment—including emotional abuse, physical abuse, emotional neglect, and physical neglect—occupies a unique position. Unlike single-incident non-interpersonal traumas (e.g., natural disasters, motor vehicle accidents), childhood maltreatment typically occurs within the caregiving system, is chronic in nature, and unfolds during the very developmental periods when attachment patterns, emotion regulation capacities, and core self-representations are being formed (9). A growing body of evidence indicates that childhood maltreatment is alarmingly prevalent among Chinese adolescents and college students. A recent network analysis involving 7,824 Chinese adolescents aged 10–19 years found substantial co-occurrence between childhood trauma and deficits in emotional awareness, and further demonstrated that maltreatment was associated with reduced perception of family support (10). In a large sample of 5,231 Chinese college students (mean age = 19.40 years), latent profile analysis revealed that approximately 27.4% of participants fell into at-risk profiles (11). The detrimental consequences of childhood maltreatment extend to the most severe forms of psychopathology: a two-wave longitudinal study of 1,286 Chinese college students found that childhood maltreatment—particularly emotional neglect and emotional abuse—was associated with elevated suicidal ideation through the chain mediating pathways of self-compassion (both positive and negative dimensions) and depressive symptoms (12). These findings collectively underscore that childhood maltreatment is not only a pervasive public health concern among Chinese youth but also one that carries lasting consequences for emotion regulation and mental health well into adolescence and emerging adulthood.

### 1.2 PTSD and PTG as co-occurring posttraumatic reactions in adolescents

In the aftermath of childhood maltreatment, adolescents may experience two contrasting yet often co-occurring psychological responses. PTSD, the most widely studied posttraumatic psychopathology, is characterized by four symptom clusters: intrusive re-experiencing, avoidance, negative alterations in cognition and mood, and alterations in arousal and reactivity (13). Among Chinese adolescents and college students exposed to childhood maltreatment, research has identified heterogeneous patterns of posttraumatic response. A latent profile analysis of 2,968 Chinese college students with childhood maltreatment history identified four distinct profiles, including a distress group characterized by high PTSD alongside moderate PTG (14). These findings corroborate the broader literature indicating that PTSD is prevalent among adolescents who have experienced trauma and frequently coexists with positive psychological changes (15).

Posttraumatic growth (PTG)—defined as positive psychological change resulting from the struggle with highly challenging life circumstances—encompasses improvements in five domains: appreciation of life, meaningful interpersonal relationships, personal strength, identification of new possibilities, and spiritual development (16). Among Chinese adolescents, PTG has been documented at notable rates (17). Furthermore, growing evidence suggests that PTSD and PTG are not mutually exclusive outcomes but rather distinct psychological processes that can coexist within the same individual (18–20). This pattern underscores a critical insight for adolescent mental health: posttraumatic responses among youth are complex and heterogeneous, and understanding the mechanisms that differentiate adaptive from maladaptive trajectories is essential.

### 1.3 Social support as a pivotal environmental resource

The theoretical frameworks that guide trauma research have increasingly acknowledged the critical role of social resources in shaping posttraumatic outcomes among adolescents. The three-phase process model of PTSD and PTG posits that social support, as a key environmental factor, exerts its influence on posttraumatic reactions by shaping how individuals regulate their emotions during the coping phase following trauma exposure (21). In the emergency phase immediately after trauma, social support ensures survivors’ physical and psychological safety; in the coping phase, it facilitates the transformation of maladaptive emotion regulation strategies into adaptive ones; and in the reaction phase, it continues to buffer against entrenchment of PTSD while consolidating cognitive and emotional gains associated with PTG.

Consistent with this theoretical framework, substantial empirical evidence links social support to both PTSD and PTG among adolescents. Perceived social support—that is, the subjective experience of being valued, understood, and cared for by one’s interpersonal network—has been shown to buffer against PTSD symptom severity across diverse trauma-exposed populations (22, 23). Concurrently, social support has been consistently identified as one of the most robust predictors of PTG (24). A study among Chinese adolescents after the Ya’an earthquake demonstrated that social support predicted PTG through self-compassion, highlighting the important role of intrapsychic mechanisms in translating environmental resources into psychological growth (25).

However, an emerging body of research has complicated the uniformly positive portrayal of social support in the trauma literature (26). Particularly when trauma is interpersonal in nature and support providers overlap with or resemble the source of the original trauma—perceived social support may paradoxically amplify rather than attenuate psychological distress. Empirical evidence for this counterintuitive pattern has been documented in trauma-exposed populations, such as disaster survivors, where interpersonal support was found to paradoxically exacerbate post-relocation adjustment difficulties rather than buffer against them (27).

Consistent with this emerging evidence, recent research among Chinese adolescents found that peer and teacher support paradoxically amplified rather than attenuated the association between adverse childhood experiences and psychological need frustration, suggesting that the buffering role of social support may be context-dependent (28). Indeed, the protective function of social support may be bounded by the level of cumulative stress exposure. Among university students, social support was found to buffer against the negative impact of stressful life events on academic performance only when stress levels were relatively low; when life event stress accumulated to higher levels, the protective effect of social support diminished, and students with high social support were still likely to experience poor academic outcomes (29). This suggests that while social support remains a resilience factor, its efficacy is not unconditional—particularly under conditions of elevated cumulative stress.

For adolescents with histories of childhood maltreatment, this paradox is structurally embedded: the very individuals who constitute their social support network (parents, caregivers, peers) may also have been the source of the original traumatic violation. Recent evidence on Chinese adolescents confirms that childhood maltreatment, especially emotional and physical neglect, is associated with reduced family support, which in turn impairs emotion regulation capacity (10). This suggests that the relationship between social support and posttraumatic outcomes among maltreated adolescents may be more complex than previously assumed, and that the mechanisms through which social support operates deserve careful empirical scrutiny.

### 1.4 Self-compassion as an emotion regulation strategy in adolescence

One promising mechanism that may clarify both the protective and the potentially paradoxical effects of social support is self-compassion. Self-compassion, as conceptualized by Neff (30, 31), is a multifaceted construct comprising three core components: self-kindness (treating oneself with warmth and understanding in the face of suffering, rather than harsh self-criticism), common humanity (recognizing that pain and imperfection are part of the shared human experience, rather than feeling isolated), and mindfulness (holding painful thoughts and emotions in balanced awareness, rather than over-identifying with them). Each component has a negative counterpart—self-judgment, isolation, and over-identification, respectively—and factor-analytic work has demonstrated that the positive and negative dimensions of self-compassion are empirically distinguishable and can exert differential effects on psychological outcomes (32, 33).

Critically for the present study, self-compassion can be conceptualized as an emotion regulation strategy (34, 35). Emotion regulation refers to the processes by which individuals influence which emotions they experience, when they experience them, and how they express them (36). Self-compassion, rather than altering the objective features of a stressful situation, changes how the individual relates to the painful emotions elicited by that situation. By responding to suffering with kindness rather than self-criticism, by framing distress within the context of shared humanity rather than private failure, and by mindfully acknowledging pain without being consumed by it, the self-compassionate individual engages in adaptive emotion regulation that has been shown to reduce the intensity and duration of negative affect (37) and to promote psychological flexibility (38). In a recent study, difficulties in emotion regulation were found to mediate the relationship between early dysfunctional schemas and emotional dependence, suggesting that emotion regulation may serve as a general mechanism linking early adverse experiences to later maladaptive outcomes (39). In the context of maltreatment-related trauma, self-compassion has been specifically identified as an adaptive emotion regulation strategy that may help survivors cope with the enduring emotional consequences of early adversity (40, 41).

The relevance of self-compassion for adolescent mental health is particularly pronounced. Adolescence is a period characterized by heightened self-consciousness, increased social comparison, and elevated risk for self-criticism and shame (42). Self-compassion interventions specifically designed for adolescents have shown promise in reducing stress, anxiety, depression, and self-criticism, while improving emotional well-being (43, 44). A growing literature demonstrates that self-compassion protects adolescents from the adverse effects of social media, peer victimization, early life trauma, and other threats to psychological well-being (45, 46). For instance, a large-scale longitudinal study among adolescents found that self-compassion, along with perceived social support, mediated the effects of emotional abuse on both non-suicidal self-injury and bullying, confirming the central role of self-compassion as a linking mechanism between interpersonal adversity and maladaptive outcomes (47). However, the evidence base on self-compassion in the context of adolescent trauma and maltreatment—particularly regarding its differential associations with co-occurring PTSD and PTG—remains relatively limited, representing a gap in the literature that the present study aims to address.

### 1.5 The present study

Integrating the theoretical and empirical perspectives reviewed above, the present study examined whether positive and negative dimensions of self-compassion mediate the associations between perceived social support and both PTSD and PTG among adolescents with histories of childhood maltreatment. Late adolescence represents a particularly vulnerable yet formative period for emotion regulation development, as the demands of identity exploration, academic pressures, and restructuring of peer and family relationships intensify, while the prefrontal neural systems that support regulatory capacity are still maturing (1, 2). Consequently, understanding how social resources and intrapsychic emotion regulation strategies jointly shape posttraumatic trajectories during this stage is essential for advancing adolescent mental health. Guided by the three-phase process model of PTSD and PTG (21) and by the emotion regulation framework of self-compassion (33, 34), we advanced the following hypotheses: (1) perceived social support would be negatively associated with PTSD and positively associated with PTG through the mediating pathway of positive self-compassion (self-kindness, common humanity, mindfulness), representing an adaptive emotion regulation pathway; (2) perceived social support would be positively associated with PTSD through the mediating pathway of negative self-compassion (self-judgment, isolation, over-identification), representing a maladaptive emotion regulation pathway; and (3) these two pathways would operate simultaneously, accounting for the dual-edged effects of social support on posttraumatic outcomes among adolescents. The findings are expected to contribute to our understanding of how social and intrapsychic resources jointly shape posttraumatic trajectories during this critical developmental period, and to inform emotion regulation-focused interventions for trauma-exposed youth.

## 2 Materials and methods

### 2.1 Participants

Participants were undergraduate adolescent students recruited from several universities across China using a convenience sampling approach. Data collection was conducted across two waves separated by a six-month interval. At Time 1 (T1), a total of 3,235 adolescent students completed the survey. At Time 2 (T2), 2,932 participants were retained, representing a retention rate of 90.6%. Participant attrition was primarily attributable to academic grade transitions. To assess the potential impact of attrition, independent-samples t-tests were conducted comparing retained and attrited participants across all study variables. No statistically significant differences were observed between the two groups, indicating that attrition was unlikely to introduce systematic bias into the findings (see **Supplementary Table 1**).

Following the administration of the Childhood Trauma Questionnaire–Short Form (CTQ-SF) (48) and application of predetermined maltreatment cut-off criteria, a final analytic sample of 1,773 eligible participants was identified. Specifically, consistent with Tietjen et al. (49) childhood maltreatment was operationalized using the following subscale cut-off scores: emotional abuse ≥ 9, physical abuse ≥ 8, emotional neglect ≥ 10, physical neglect ≥ 8, and sexual abuse ≥ 6. This classification scheme has demonstrated satisfactory sensitivity and specificity in prior studies (49) and has been validated in Chinese samples (50). Detailed information regarding the distribution of the specific childhood maltreatment subtypes within the final analytic sample is provided in **Supplementary Table 2**. Among the 1,773 participants who met inclusion criteria, 845 (47.7%) identified as male and 928 (52.3%) as female. With respect to age, all participants were adolescents under 20 years of age, with 1,218 participants (68.7%) being 18 years or younger and 555 (31.3%) being 19 years old.

### 2.2 Procedure

Prior to data collection, ethical approval was obtained from the Institutional Review Board (IRB) of the Faculty of Psychology at Beijing Normal University (Approval No.12220085). Participants were recruited through university-wide communications distributed to students. Those who expressed willingness to participate were subsequently enrolled in the study. Before commencing the survey, all participants provided written informed consent; for students under 18 years of age, parental consent was also obtained. Participants were informed of their right to withdraw at any time without consequence, particularly if any item caused psychological discomfort. At T1, participants completed measures of childhood maltreatment, perceived social support, PSC, NSC, PTSD, and PTG. At T2, six months later, measures of PSC, NSC, PTSD symptoms, and PTG were re-administered.

### 2.3 Measures

#### 2.3.1 Childhood maltreatment

Childhood maltreatment was assessed using the Childhood Trauma Questionnaire–Short Form (CTQ-SF) (48) a well-validated instrument comprising 28 items across five subscales: physical abuse, physical neglect, emotional abuse, emotional neglect, and sexual abuse. The validated Chinese version (51) was used in the present study. Each item is rated on a 5-point Likert scale ranging from 1 (never) to 5 (very often), with higher scores indicating greater maltreatment severity. The CTQ-SF has demonstrated adequate reliability and validity in Chinese populations (52). In the present study, Cronbach’s α coefficients for the five subscales were as follows: physical abuse = .819, emotional abuse = .694, physical neglect = .146, emotional neglect = .855, and sexual abuse = .860. The comparatively low internal consistency observed for the physical neglect subscale is consistent with patterns reported in prior research using this instrument (53). As noted in methodological literature regarding trauma inventories, maltreatment experiences function as an objective events measure rather than a traditional psychological construct. Because distinct traumatic events (e.g., lacking food vs. lacking medical care) can occur independently and do not necessarily co-occur, low internal consistency may be therefore acceptable for this specific subscale.

#### 2.3.2 Social support

Perceived social support was assessed using the Multidimensional Scale of Perceived Social Support (MSPSS) (54), a 12-item instrument that evaluates social support across three sources: family, friends, and significant others. Items are rated on a 7-point Likert scale ranging from 1 (very strongly disagree) to 7 (very strongly agree), with higher scores reflecting greater perceived social support (55). In the present study, Cronbach’s α coefficients for the three subscales were: family support = .951, friend support = .964, and other support = .949.

#### 2.3.3 Self-compassion

Self-compassion was measured using Short Form Self-Compassion Scale (SCS-SF) (56). This scale consists of 12 items, yielding two dimensions: PSC (including three subscales: common humanity, self-kindness, and mindfulness) and NSC (including three subscales: isolation, self-judgment, and over-identification). Items are rated on a 5-point Likert scale ranging from 1 (almost never) to 5 (almost always). The scale has demonstrated good reliability and construct validity in Chinese populations (57). In the present study, Cronbach’s α coefficients were: T1 PSC = .917, T1 NSC = .882, T2 PSC = .948, and T2 NSC = .918.

#### 2.3.4 PTG

The posttraumatic growth subscale from the Short Form of the Posttraumatic Growth and Posttraumatic Depreciation Inventory–Expanded (PTGDI-X-SF) (58) was used to assess PTG. This 10-item subscale consists of five domains: appreciation of life, life philosophy, new possibilities, relating to others, and personal strength. Items are rated on a 5-point Likert scale from 1 (almost never) to 5 (almost always), with higher scores indicating greater PTG. In the present study, Cronbach’s α was.976 at T1 and.984 at T2.

#### 2.3.5 PTSD

PTSD was assessed using the PTSD Checklist for DSM-5 (PCL-5) (59), as adapted for Chinese populations by Zhou et al. (60). The PCL-5 is a 20-item measure assessing the four DSM-5 symptom clusters: intrusion, avoidance, negative alterations in cognition and mood, and hyperarousal. Each item is rated on a 4-point scale from 0 (not at all) to 3 (extremely), with higher total scores indicating greater symptom severity. In the present study, Cronbach’s α was.964 at T1 and.967 at T2.

### 2.4 Data analysis

To evaluate potential common method bias (CMB), we employed both Harman’s single-factor test and a more rigorous unmeasured latent method factor (ULMF) approach (61). Consistent with established criteria (62), acceptable model fit was indicated by χ²/df values between 1 and 3, CFI and TLI values ≥.90, and RMSEA values ≤.08. Given that the χ² statistic is highly sensitive to large sample sizes and is frequently inflated (63), model fit was primarily evaluated using indices less affected by sample size.

It should be noted that although we employed a two-wave longitudinal design and controlled for autoregressive effects, the mediator (self-compassion) and outcomes (PTSD and PTG) were measured concurrently at T2. Consequently, the paths are essentially cross-sectional in nature, and the indirect effects should be interpreted as prospective predictive associations rather than as true longitudinal mediation effects.

## 3 Results

### 3.1 Descriptive statistics

The results of descriptive statistics of the study variables and their intercorrelations are presented in **Table 1**. The results showed varying degrees of correlation among the study variables. Additionally, both sex and age were associated with the study variables, as such they were controlled as covariates in the subsequent analysis.

**Table 1:** The mean, standard deviation, and intercorrelation among all study variables (N = 1773).

Variables	*M* ± *SD*	1	2	3	4	5	6	7	8	9	10	11
1. Sex^a^	–	-										
2. Age^b^	–	-.091^***^	-									
3. T1 Social Support	62.764 ± (16.616)	-.107^***^	.191^***^	-								
4 T1 PSC	19.725 ± (5.340)	-.061^*^	.087^***^	.551^***^	-							
5. T2 PSC	17.045 ± (6.506)	-.018	.183^***^	.262^***^	.294^***^	-						
6. T1 NSC	16.579 ± (5.137)	-.048^*^	-.023	.211^***^	.516^***^	.068^**^	-					
7. T2 NSC	13.977 ± (5.510)	-.003	.077^**^	.095^***^	.147^***^	.67^***^	.263^***^	-				
8. T1 PTG	32.385 ± (15.531)	-.014	.064^**^	.346^***^	.327^***^	.198^***^	.097^***^	.085^***^	-			
9. T2 PTG	26.974 ± (15.320)	-.008	.097^***^	.224^***^	.216^***^	.411^***^	.044	.256^***^	.342^***^	-		
10. T1 PTSD	40.968 ± (16.916)	-.009	.008	-.155^***^	.014	-.014	.343^***^	.159^***^	.117^***^	.045^**^	-	
11. T2 PTSD	35.350 ± (15.648)	-.003	.051^**^	-.056^*^	.010	.193^***^	.216^***^	.399^***^	.036	.238^***^	.416^***^	-

^a^0 = male, 1 = female; ^b^1 = under 18 years, 2 = 19 years; PSC, positive self-compassion; NSC, negative self-compassion; PTSD, posttraumatic stress disorder; PTG, posttraumatic growth. ^*^p <.05, ^**^p <.01, ^***^p <.0.

### 3.2 SEM analysis

Six steps were conducted for the SEM analysis: First, we evaluated the measurement structure of self-compassion. Specifically, confirmatory factor analyses (CFAs) were conducted to compare a one-factor model of the Short Form Self-Compassion Scale (SCS-SF) with a two-factor model distinguishing positive self-compassion (PSC) and negative self-compassion (NSC). The CFA results indicated that the two-factor model of the SCS-SF demonstrated substantially better fit than the one-factor model (CFI = .983 vs.747; TLI = .968 vs.578; RMSEA = .097 vs.350), supporting the distinction between PSC and NSC.

Second, we evaluated the longitudinal measurement invariance for PSC/NSC, PTSD, and PTG across the two waves to ensure that the latent constructs were measured equivalently over time. The sequential longitudinal measurement invariance analyses supported metric invariance for PSC/NSC, PTSD, and PTG across the two waves (see **Supplementary Table 3**), indicating that these latent constructs were measured consistently over time.

Third, we estimated a measurement model consisting of 33 observed variables and nine latent variables (social support at T1, PSC and NSC at T1 and T2, PTG and PTSD at T1 and T2). Results indicated that the model fit the data well: χ²(459) = 3537.415, *p* < 0.001, χ²/*df* = 7.707, CFI = 0.969, TLI = 0.964, and RMSEA (90% CI) = 0.054 (0.052-0.056). Notably, while the χ² and the χ²/*df* results were sensitive to our large sample size, the evidence of a good model fit was primarily supported by the robust alternative indices (CFI, TLI, and RMSEA). Our results of Harman’s single-factor test indicated no significant CMB with the variance accounted for by the first factor without rotation of 16.77% (< 40%) (61). Furthermore, the ULMF model demonstrated model fit indices as follows: χ² = 3904.428, χ²/df = 7.367, CFI = 0.966, TLI = 0.962, RMSEA = 0.053. Compared to the original measurement model, the inclusion of the method factor did not substantially improve the model fit (ΔCFI = 0.003, ΔRMSEA = 0.001, both < 0.01). These results indicated no severe CMB in the current data.

Fourth, a direct-effect model was estimated to examine the prospective associations between T1 social support and T2 PTSD and PTG after controlling for the autoregressive effects of PTSD and PTG. The model demonstrated acceptable fit (χ²(220) = 2640.683, p < 0.001, CFI = .965, TLI = .960, RMSEA = .069). T1 social support significantly predicted higher T2 PTG (β = 0.051, SE = 0.011, 95% CI = [0.035, 0.072], *p* < 0.001) but was not significantly associated with T2 PTSD (β = 0.002, SE = 0.015, 95% CI = [-0.022, 0.025], *p* = 0.889) after controlling for autoregressive effects.

**Figure 1:**
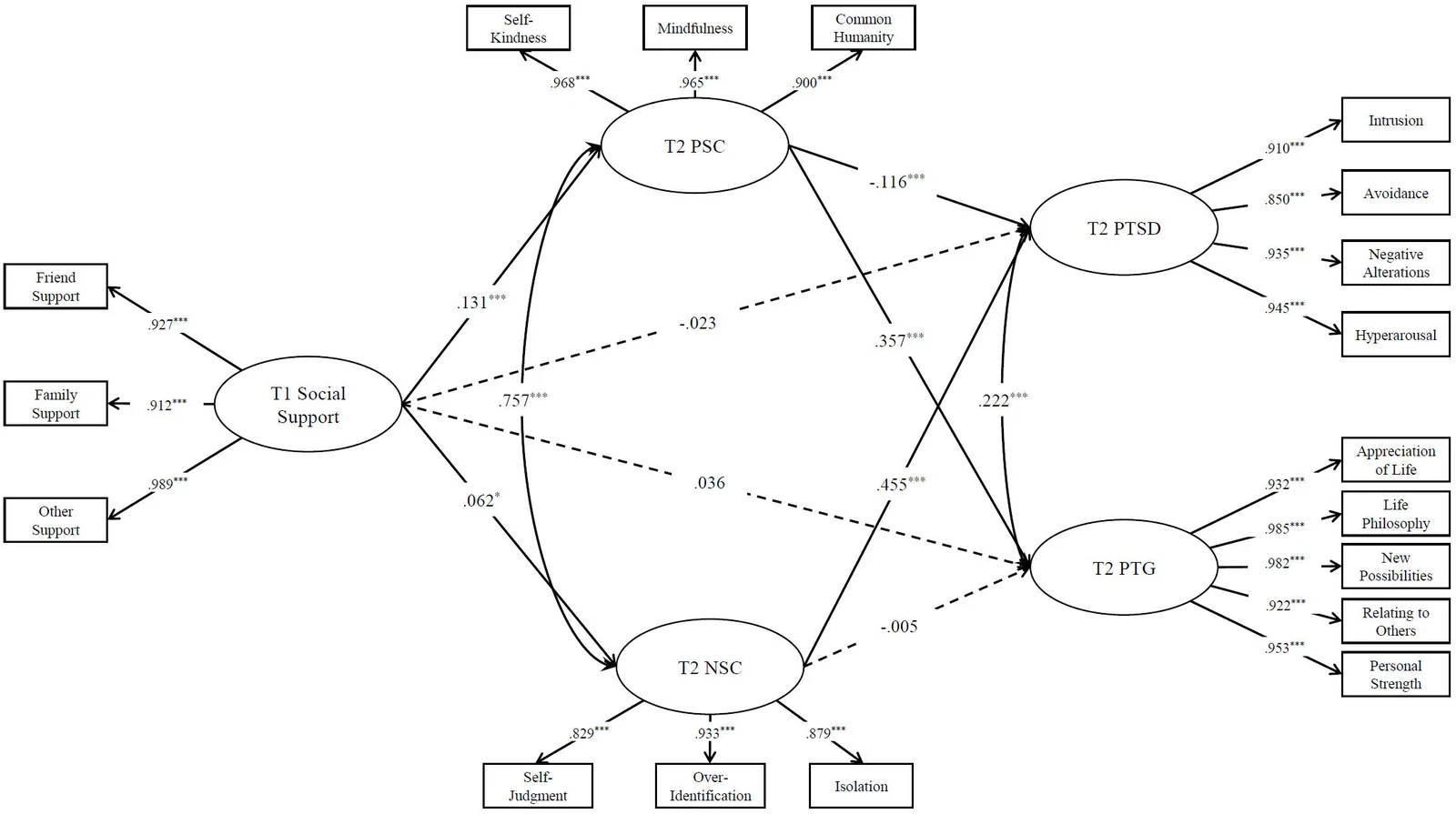
Indirect effect models. Standardized parameters are reported. Covariates are not depicted for simplicity. PSC, positive self-compassion; NSC, negative self-compassion; PTG, posttraumatic growth; PTSD, posttraumatic stress disorder. ^*^p <.05, ^***^p <.001.

Fifth, the indirect effect model including social support, PSC, NSC, PTG, and PTSD was estimated to examine the hypothesized mediation model (see **Figure 1**). PSC and NSC, as well as PTG and PTSD, were allowed to covary. Autoregressive effects were controlled by specifying predictive paths from T1 PSC/NSC to T2 PSC/NSC and from T1 PTG/PTSD to T2 PTG/PTSD. The indirect effect model demonstrated good fit to the data, χ²(532) = 3135.742, *p* <.001, χ²/df = 5.894, CFI = .964, TLI = .960, and RMSEA = .053(0.051-0.054). Social support positively predicted PSC (β = 0.131, SE = 0.010, 95% CI = [0.081, 0.182], *p* <.001) and NSC (β = 0.062, *SE* = 0.009, 95% CI = [0.008, 0.116], *p* = .043). PSC positively predicted PTG (β = 0.357, *SE* = 0.051, 95% CI = [0.293, 0.423], *p* <.001) and negatively predicted PTSD (β = -0.116, *SE* = 0.092, 95% CI = [-0.162, -0.069], *p* <.001), whereas NSC positively predicted PTSD (β = 0.455, *SE* = 0.092, 95% CI = [0.404, 0.506], *p* <.001) but was not associated with PTG. To further examine the robustness of the hypothesized temporal ordering and address the potential bidirectionality between PSC/NSC and PTSD/PTG, an alternative structural model was estimated by additionally specifying reverse cross-lagged paths from T1 PTSD and PTG to T2 PSC and NSC while retaining all hypothesized paths in the original mediation model. In this model, two reverse paths reached statistical significance: T1 PTSD positively predicted T2 NSC (β = 0.111, *p* < 0.001), T1 PTG positively predicted T2 PSC (β = 0.098, *p* < 0.001). These reverse coefficients (βs = 0.060-0.111) were comparable in magnitude to the paths from social support to self-compassion (βs = 0.062-0.131), while smaller than the paths from self-compassion to posttraumatic outcomes (βs = 0.116-0.455).

Sixth, the results of significant indirect paths were shown in **Table 2**. Results indicated that social support positively predicted PSC, which in turn, positively predicted PTG and negatively predicted PTSD. Besides, the bootstrapping confidence interval indicated a marginally significant indirect trend that social support positively predicted PTSD via its positive prediction of NSC. Yet, the lower bound of confidence interval is small (0.003) and the p-value did not reach traditional significance levels ((p = .067, marginally significant). Thus, this pathway should be interpreted with caution. Moreover, even the statistically significant indirect pathways via positive self-compassion yielded relatively small standardized coefficients (𝛽=0.047 for PTG and 𝛽=−0.015 for PTSD), suggesting that the practical or clinical significance of these effects should be interpreted with appropriate caution.

**Table 2:** Standardized indirect path coefficients and bias-corrected bootstrapping test (N = 1773).

Indirect paths	Standardized β	SE	Standardized 95% CI		p
			Low	High	
**T1 Social Support → T2 PSC → T2 PTG**	**0.047**	**0.012**	**0.029**	**0.068**	**<0.001**
**T1 Social Support → T2 PSC → T2 PTSD**	**-0.015**	**0.005**	**-0.026**	**-0.008**	**0.001**
T1 Social Support → T2 NSC → T2 PTG	0.000	0.003	-0.004	0.004	0.763
**T1 Social Support → T2 NSC → T2 PTSD**	**0.028**	**0.015**	**0.003**	**0.054**	**0.067**

PSC, positive self-compassion; NSC, negative self-compassion; PTG, posttraumatic growth; PTSD, posttraumatic stress disorder. Significant and marginal significant indirect paths were bold.

## 4 Discussion

The present study examined the mediating roles of positive and negative self-compassion in the links between perceived social support and both PTSD and PTG among adolescents with histories of childhood maltreatment. Using a two-wave longitudinal design, we identified two distinct pathways through which social support was associated with posttraumatic outcomes. The adaptive pathway operated through positive self-compassion: social support predicted greater positive self-compassion, which in turn was associated with lower PTSD and higher PTG. The maladaptive pathway operated through negative self-compassion: social support predicted greater negative self-compassion, which in turn was associated with elevated PTSD. These findings extend the literature on adolescent posttraumatic adjustment by demonstrating that self-compassion functions as a dual-edged emotion regulation mechanism linking social resources to divergent psychological outcomes.

### 4.1 The adaptive pathway through positive self-compassion

The finding that social support predicted lower PTSD and greater PTG indirectly through positive self-compassion aligns with the emotion regulation framework of self-compassion (34, 35). Among adolescents who perceived greater support from family, friends, and significant others, the capacity to treat themselves with kindness, recognize their suffering as part of shared human experience, and hold painful emotions in mindful awareness was enhanced. This is consistent with theoretical models positing that social support operates not only as a direct buffer against distress but also as a relational context that cultivates intrapsychic emotion regulation resources (21). From a developmental perspective, adolescence is a period during which the capacity to internalize supportive external relationships into self-soothing capacities is particularly salient (6, 7). For adolescents with maltreatment histories, whose early caregiving experiences were characterized by threat or neglect, supportive relationships during late adolescence may serve as corrective experiences that facilitate the development of self-compassion as an adaptive emotion regulation strategy (40).

Once activated, positive self-compassion was associated with both lower PTSD and higher PTG. The inverse association with PTSD is consistent with a substantial body of evidence demonstrating that self-compassion protects against posttraumatic psychopathology (15, 64). By responding to trauma-related distress with self-kindness rather than self-blame, adolescents may interrupt the shame and negative self-appraisals that maintain PTSD symptomatology according to cognitive models of the disorder (65). The positive association with PTG supports the proposition that self-compassion is not merely a buffer against distress but an active catalyst for growth. Adolescents who approach their suffering with mindful acceptance and a sense of common humanity may be better positioned to engage in the deliberate cognitive processing—reappraisal, meaning-making, narrative reconstruction—that underlies PTG (16). Consistent with this, prior research has demonstrated that self-compassion predicts PTG among adolescents following both natural disasters (25) and childhood maltreatment (15).

### 4.2 A potential maladaptive pathway through negative self-compassion

A more novel contribution of this study is the identification of a potential maladaptive pathway through which social support was associated with elevated PTSD via heightened negative self-compassion. While this finding should be interpreted cautiously given the p-value and CI, it offers a preliminary observation that is broadly consistent with the paradoxical pattern documented in prior trauma research, where interpersonal support sometimes fails to buffer—or may even intensify—psychological distress (27). This suggests that among maltreated adolescents, perceived social support may, under certain circumstances, be associated with elevated distress through heightened negative self-compassion. For adolescents with childhood maltreatment histories, the receipt or perception of support may, in some cases, activate self-judgment, felt isolation, and over-identification with pain—the core components of negative self-compassion.

One interpretation of this finding concerns the relational paradox inherent in interpersonal trauma. In families where maltreatment has occurred, caregivers—who are simultaneously the prototypical sources of attachment security and the perpetrators of harm—constitute the child’s primary support system. When social support is perceived from figures who resemble or evoke the original source of trauma, the experience of receiving care may trigger trauma-related schemas of vulnerability and unworthiness. Rather than soothing distress, the support may intensify self-criticism (“I must be weak to need this help”) and felt isolation (“no one can truly understand what I have experienced”). This interpretation is consistent with recent evidence linking childhood maltreatment to reduced family support and impaired emotion regulation among Chinese adolescents (10). The finding that negative self-compassion robustly predicted PTSD—with a standardized coefficient larger than that of positive self-compassion—is consistent with meta-analytic evidence that the negative components of self-compassion are more strongly associated with psychopathology than positive components are with well-being (32). However, this potential maladaptive pathway should be interpreted with the caveat of empirical bidirectionality. Specifically, our alternative structural model revealed a significant prospective effect from T1 PTSD to T2 negative self-compassion. It indicates that posttraumatic stress and negative self-compassion may operate in a recursive, mutually reinforcing feedback loop over time rather than through a strictly unidirectional pathway.

In this regard, cultural factors may further amplify the paradoxical effects of family support. In collectivistic cultural contexts such as China, family relationships are characterized by high interdependence, strong expectations of filial piety, and the internalization of family obligations as central to self-worth (66). For maltreated adolescents, perceived family support may thus carry ambivalent meanings—care and validation may coexist with the activation of traumatic memories and heightened self-critical standards associated with filial expectations. When support is received from family members who are also associated with past maltreatment, the resulting cognitive dissonance may intensify feelings of isolation, self-judgment, and over-identification with distress, rather than soothing them. Moreover, mental health stigma prevalent in Chinese cultural contexts may further reduce adolescents’ willingness to openly seek or effectively internalize social support, potentially undermining its protective function (67). These cultural dynamics may help explain why, in our sample of Chinese late adolescents, the negative self-compassion pathway showed a marginal trend toward heightened PTSD, even though social support simultaneously activated the adaptive positive self-compassion pathway.

### 4.3 Differential associations with PTSD and PTG and the absence of direct effects

The specificity of negative self-compassion’s association with PTSD, and its lack of significant association with PTG, suggests that self-judgment, isolation, and over-identification may represent forms of maladaptive emotion regulation that trap individuals in cycles of distress without facilitating growth. Unlike positive self-compassion, which enables the survivor to approach trauma-related material with an attitude of openness and curiosity, negative self-compassion may promote experiential avoidance and self-critical rumination—processes that maintain PTSD while inhibiting the meaning-making that underlies PTG (15, 68). This differential pattern reinforces the importance of examining positive and negative self-compassion as distinct dimensions rather than as a unitary construct.

The direct effects of social support on PTSD and PTG showed different patterns. Specifically, the null total effect of social support on PTSD can be explained by two opposing and almost canceling indirect pathways: a protective pathway through positive self-compassion and a potential risk pathway through negative self-compassion. In contrast, the association between social support and PTG remained partially direct after accounting for self-compassion, indicating that additional mechanisms beyond self-compassion may be involved in facilitating growth. These findings suggest that, among maltreated adolescents, social support may reduce posttraumatic stress primarily by fostering adaptive self-relating and reducing self-critical responses. In contrast, the beneficial effect of social support on posttraumatic growth appears to operate through both self-compassion and additional pathways beyond self-compassion. From a clinical perspective, these findings suggest that enhancing social support alone may be insufficient for alleviating posttraumatic stress unless such support is internalized into adaptive self-relating patterns (21). At the same time, social support may also facilitate posttraumatic growth through other mechanisms, such as strengthening interpersonal resources, promoting meaning-making, or increasing opportunities for positive developmental experiences.

### 4.4 Implications for adolescent mental health

These findings carry several implications for emotion regulation-focused interventions with trauma-exposed adolescents. First, interventions that directly cultivate positive self-compassion—such as mindful self-compassion programs designed for adolescents (43)—may be particularly effective in reducing PTSD and promoting PTG, as they target the very mechanism through which social support exerts its adaptive effects. The particular potency of self-compassion as an intervention target is underscored by recent evidence from a machine-learning study of Chinese adolescents, which found that among multiple positive psychological traits—including mindfulness, self-compassion, and gratitude—self-compassion emerged as the second most important predictor of CPTSD symptoms (69). This suggests that self-compassion, relative to other positive traits, may be a particularly potent leverage point for intervention among trauma-exposed youth. Second, given the potential maladaptive pathway through negative self-compassion, clinicians should assess not only the presence of social support but also how adolescents interpret and internalize the support they receive. For some adolescents, particularly those whose trauma involves caregivers, helping them differentiate between genuinely supportive relationships and those that evoke trauma-related schemas may be a necessary precursor to compassion-focused work (41). However, because this pathway did not reach conventional statistical significance, specific clinical recommendations based on this trend would be premature. Third, our findings highlight the importance of addressing self-criticism and felt isolation as distinct clinical targets. Compassion-focused therapy approaches that explicitly target shame and self-criticism may be especially indicated for this population (64). Nevertheless, given the relatively small indirect effect sizes observed in this study, the clinical implications should be considered preliminary, and the practical significance of these effects awaits further empirical validation in controlled intervention trials. Finally, the inclusion of participants was based on established CTQ-SF cut-off scores. Given the inherently low internal consistency of the physical neglect subscale as an event-based measure, our inclusion may introduce some classification bias into our sample definition, potentially excluding adolescents with atypical but severe single-incident neglect experiences.

### 4.5 Limitations and future directions

Several limitations warrant consideration. First, all measures were self-report, introducing potential shared method variance; future studies could incorporate behavioral, physiological, or multi-informant assessments. Second, although our two-wave design provided prospective evidence for the hypothesized pathways, denser assessments with three or more time points would allow for a more rigorous examination of reciprocal dynamics and true temporal mediation among the variables. Third, our sample comprised Chinese late adolescents; cross-cultural research is needed to determine whether these pathways generalize across cultural contexts. Fourth, although we used childhood maltreatment as a sample inclusion criterion to ensure a homogeneous trauma-exposed sample, we did not formally examine maltreatment severity, maltreatment type, or source of social support as potential moderators of the hypothesized mediation pathways. It is plausible that adolescents with more severe maltreatment may exhibit stronger associations between perceived social support and negative self-compassion, or that different maltreatment types may differentially moderate the links between self-compassion and posttraumatic outcomes. Additionally, the source of social support—family versus friends—may interact with the nature of maltreatment. Future studies with larger and more diverse samples are needed to systematically test these potential moderating effects and to clarify the boundary conditions under which social support serves as a buffer versus a risk factor among maltreated adolescents. Fifth, other emotion regulation strategies beyond self-compassion may also mediate the social support–posttraumatic outcomes link and warrant investigation in future research. It is also worth noting that all study variables—PSC, NSC, PTG, and PTSD—declined from T1 to T2. This synchronous decrease may be partially attributable to retest effects, regression to the mean, or the transition from the early to late semester. Although the autoregressive paths in our model statistically controlled for baseline levels, thereby reducing the influence of such general trends on the predictive relationships of interest, readers should be aware of this pattern when interpreting the cross-time findings. In addition, although our sample falls within the WHO-defined adolescent age range (10–19 years), participants were exclusively 18- to 19-year-old university students, representing a relatively narrow segment of late adolescence. This limits the generalizability of our findings to younger adolescents (e.g., middle school students) or to older individuals in emerging adulthood (ages 20–25). Future studies should examine whether the observed self-compassion pathways replicate across broader age ranges within adolescence.

### 4.6 Conclusion

This study demonstrates that among adolescents exposed to childhood maltreatment, perceived social support is associated with posttraumatic outcomes through two distinct self-compassion pathways. The adaptive pathway—social support predicting lower PTSD and greater PTG through positive self-compassion—represents a health-promoting emotion regulation mechanism. A potential, albeit marginally significant, maladaptive pathway-–social support possibly predicting elevated PTSD through negative self-compassion-–illuminates the complexity of social support for this population and underscores the need for further investigation. These findings advance understanding of how social and intrapsychic resources jointly shape adolescent posttraumatic adjustment and highlight self-compassion as a promising intervention target for trauma-exposed youth.

It is also important to note that the indirect effect sizes identified in this study were relatively small. While statistically significant for the positive self-compassion pathway, the practical or clinical significance of these effects requires further investigation. Future studies with larger samples, longer follow-up periods, and experimental or intervention designs are needed to determine whether self-compassion-focused interventions yield clinically meaningful changes in posttraumatic outcomes among maltreated adolescents.

## Data Availability

The raw data supporting the conclusions of this article will be made available by the authors, without undue reservation.
