# Supplementary material for: Does social support always help? Emotion regulation pathways through self-compassion in maltreated adolescents’ PTSD and growth
Source: Front Psychiatry. 2026 Aug 27;17:1889693. doi: 10.3389/fpsyt.2026.1889693 (PMC13558192; doi:10.3389/fpsyt.2026.1889693)
Supplement: Supplementary file 1 [file Table1.docx]

**Table S1**

*Attrition randomness check*

| Variable | *M* ± *SD* / *n* (%) | | Test statistic | *p* |
| --- | --- | --- | --- | --- |
|  | Retained | Attrition |  |  |
| Age |  |  | *χ*²(1) = 2.57 | 0.11 |
| 18 years old or younger | 2028 (69.17%) | 196 (64.69%) |  |  |
| 19 years old | 904 (30.83%) | 107 (35.31%) |  |  |
| Sex |  |  | *χ*²(1) = 2.39 | 0.12 |
| Male | 1258 (42.91%) | 144 (47.52%) |  |  |
| Female | 1674 (57.09%) | 159 (52.48%) |  |  |
| Social Support | 66.92 ± 15.69 | 64.91 ± 18.28 | *t*(349.51) = -1.85^a^ | 0.66 |
| PSC | 20.47 ± 5.17 | 20.12 ± 5.69 | *t*(3233) = -1.13 | 0.26 |
| NSC | 16.18 ± 5.12 | 16.32 ± 5.89 | *t*(350.66) = 0.40^a^ | 0.66 |
| PTG | 34.41 ± 15.90 | 33.68 ± 16.68 | *t*(3233) = -0.75 | 0.45 |
| PTSD | 38.44 ± 16.25 | 36.58 ± 15.75 | *t*(3233) = 0.57 | 0.06 |

*Note.* ^a^ Equal variances were not assumed due to a significant Levene’s test for equality of variances, and Welch’s t-test results are reported with adjusted degrees of freedom. PSC = positive self-compassion, NSC = negative self-compassion, PTSD = posttraumatic stress disorder, PTG = posttraumatic growth.

**Table S2**

*Mean, Standard Deviation, and Number and Proportion of Participants Reaching the Cut-off Score for Childhood Maltreatment Experiences*

|  | **Pre-screening (N = 2932)** | | **Post-screening (N = 1773)** | | **Number of Reaching Cut-off** | **% of Total Sample** | **% of Maltreated Sample** |
| --- | --- | --- | --- | --- | --- | --- | --- |
|  | **M** | **SD** | **M** | **SD** |  |  |  |
| 1. Physical abuse | 5.558 | 1.638 | 5.828 | 2.034 | 213 | 7.3% | 12.0% |
| 2. Physical neglect | 7.690 | 2.978 | 9.254 | 2.901 | 1230 | 42.0% | 69.4% |
| 3. Emotional abuse | 6.675 | 2.364 | 7.323 | 2.782 | 490 | 16.7% | 27.6% |
| 4. Emotional neglect | 9.806 | 5.036 | 12.172 | 5.156 | 1260 | 43.0% | 71.1% |
| 5. Sexual abuse | 5.243 | 1.161 | 5.341 | 1.426 | 277 | 9.5% | 15.6% |

**Table S3**

*Longitudinal Measurement Invariance of PSC/NSC, PTSD, and PTG*

| **Construct** | **Model** | **χ²** | **df** | **CFI** | **TLI** | **RMSEA (90% CI)** | **ΔCFI** | **ΔRMSEA** |
| --- | --- | --- | --- | --- | --- | --- | --- | --- |
| PSC/NSC | Configural | 297.223 | 42 | .980 | .968 | .059 (.052–.065) | — | — |
|  | Metric | 309.005 | 46 | .979 | .970 | .057 (.051–.063) | .001 | -.002 |
| PTSD | Configural | 193.642 | 15 | .979 | .960 | .082 (.072–.092) | — | — |
|  | Metric | 346.760 | 18 | .970 | .952 | .090 (.085–.094) | .009 | .008 |
| PTG | Configural | 323.716 | 29 | .972 | .957 | .076 (.068–.083) | — | — |
|  | Metric | 358.588 | 33 | .969 | .958 | .075 (.068–.082) | .003 | -.001 |

*Note*: PSC = positive self-compassion, NSC = negative self-compassion, PTSD = posttraumatic stress disorder, PTG = posttraumatic growth.
